# Perceptual Grouping of Object Contours Survives Saccades

**DOI:** 10.1371/journal.pone.0021257

**Published:** 2011-06-21

**Authors:** Maarten Demeyer, Peter De Graef, Karl Verfaillie, Johan Wagemans

**Affiliations:** Laboratory of Experimental Psychology, University of Leuven (K.U. Leuven), Leuven, Belgium; University of Alberta, Canada

## Abstract

Human observers explore scenes by shifting their gaze from object to object. Before each eye movement, a peripheral glimpse of the next object to be fixated has however already been caught. Here we investigate whether the perceptual organization extracted from such a preview could guide the perceptual analysis of the same object during the next fixation. We observed that participants were indeed significantly faster at grouping together spatially separate elements into an object contour, when the same contour elements had also been grouped together in the peripheral preview display. Importantly, this facilitation occurred despite a change in the grouping cue defining the object contour (similarity versus collinearity). We conclude that an intermediate-level description of object shape persists in the visual system across gaze shifts, providing it with a robust basis for balancing efficiency and continuity during scene exploration.

## Introduction

Humans scan their surroundings through the execution of a series of fast eye movements, known as saccades. These gaze shifts are necessary because only a small central part of the retina, the fovea, supports high-resolution processing of the visual input. Prior to the saccade an analysis of the peripheral visual field needs to be performed, however, both to make an informed decision on which object to foveate next and to accurately program the eye movement itself. This implies that each saccade target object has been processed up to at least some degree both in presaccadic and in postsaccadic vision. The question then is what kind of information extracted from the peripheral pre-processing of an object persists throughout the eye movement, so as to maintain perceptual continuity and make the object's subsequent re-analysis in the fovea more efficient [1].

A considerable body of evidence has suggested that transsaccadic visual memory for objects is sparse. Consequently, any effect that a presaccadic peripheral glimpse of an object could have on the postsaccadic perceptual analysis of the same object would pertain to its coarse or structural properties, rather than its visual detail [2]–[9]. In recent work, however, we have argued that objects are remembered to a greater degree of detail across gaze shifts than was previously theorized [10]–[13].

But transsaccadic visual memory cannot be *too* extensive either. Given the lower acuity in the peripheral visual field, fine-grained preview information cannot be registered reliably. After all, obtaining visual detail is the very reason why observers make eye movements. There seems to be little benefit then in retaining large quantities of unreliable information across saccades, when within tens of milliseconds a much improved sample of the same object will become available in the fovea of the visual field. Moreover, it is unclear how perceptual continuity across gaze shifts could be achieved when subsequent visual inputs on the same object differ greatly in spatial resolution. Sparse-memory theories of transsaccadic perception [7] suggest that this problem does not arise when only a minimal description of the preview is retained in the visual system across a saccade. An alternate view however [14]–[15] holds that transsaccadic vision operates at more intermediate stages of visual processing. This way, it can discard visual information up to a level where it becomes independent of the local feature information that is being distorted by low spatial resolution, while still retaining detailed information on the overall object shape.

The brain does indeed construct such mid-level representations on the basis of its visual input, inducing a perception of detailed object shape or structure beyond what is contained within the pixel information of the stimulus image. This can be demonstrated through the well-known phenomenon of perceptual grouping, that is, the visual system's behavior of treating spatially separate local information as belonging together on the basis of being close, similar, or in good continuation of one another [16]–[18]. A clearly segregated *Gestalt* defining an object shape can then be evoked even from a sparse or noisy display, where little or no local feature information is by itself indicative of the presence of a contour.

**Figure 1:**
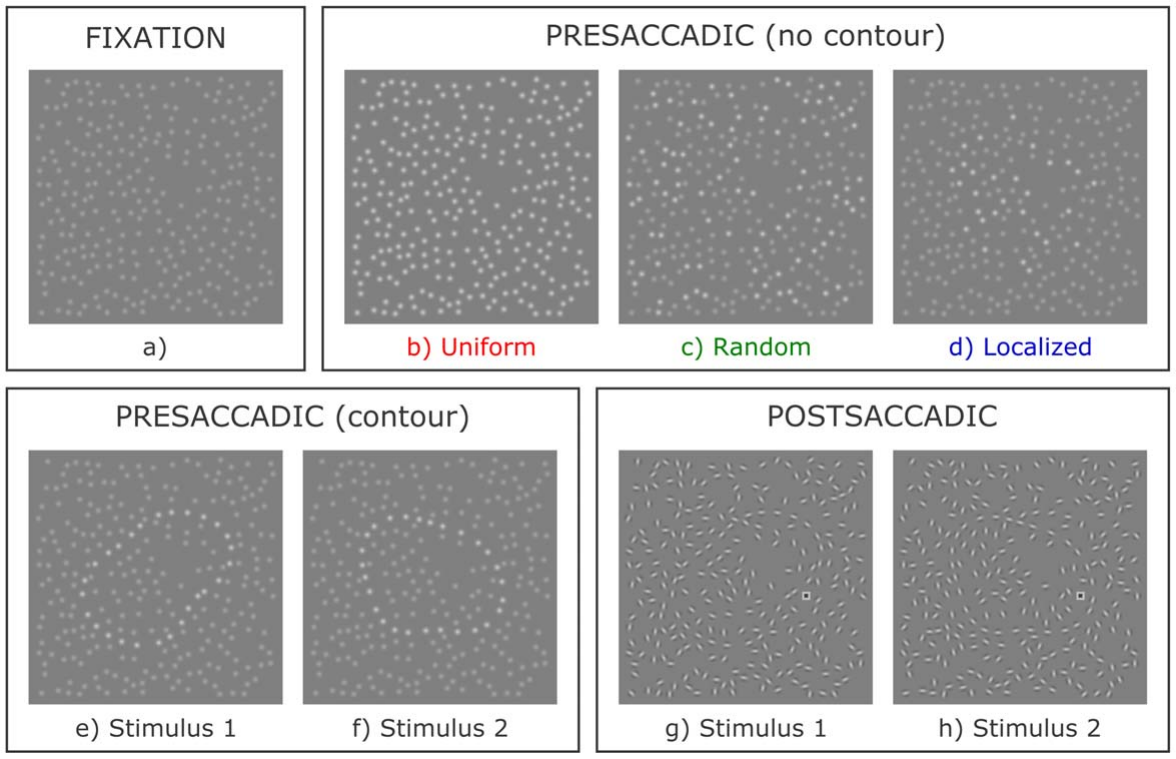
An example stimulus set. a) Fixation display; b) Uniform neutral preview display c) Random neutral preview display; d) Localized neutral preview display; e) Luminance-defined preview display showing stimulus 1; f) Luminance-defined preview display showing stimulus 2; g) Orientation-defined postsaccadic display showing stimulus 1; h) Orientation-defined postsaccadic display showing stimulus 2. The subject's task was to locate as quickly as possible the small square as being either outside (as in panel g) or inside the object contour (as in panel h).

In the present study, we used perceptual grouping displays to test the aforementioned hypothesis that transsaccadic vision makes use of intermediate-level representations of object shape. Previous studies [14], [19] have used adaptation paradigms to demonstrate that the same mid-level neurons process the same spatiotopic locations across eye movements, but have not aimed to show a functional advantage to this neuronal architecture. Specifically, we investigated whether observers would be faster at grouping together a foveally presented object contour following a saccade, when presented with a peripheral preview of an identically grouped contour at the same spatiotopic location prior to the eye movement. Critically, the grouping cue changed during the saccade, from luminance similarity of Gaussian elements in the preview display (e.g. Figure 1e) to collinearity of Gabor elements in the postsaccadic display (e.g. Figure 1g). This precluded local image features from playing a role. After executing the saccade, while viewing the collinearity-defined display, subjects were instructed to respond as fast as possible whether a small square target was located inside or outside the object's closed contour. This required them to segregate the object shape from the background, and the inside from the outside elements, through perceptual grouping.

Critical manipulations were not made in the postsaccadic display, but in the extent to which the preceding peripheral preview resembled it. The preview itself never allowed the preparation of a response because it did not contain the target square. It could only affect the response through its effect on the speed of the postsaccadic contour grouping process, which we did assume to directly influence the manual response speed. Compared to a **Uniform** neutral preview condition (Figure 1b), we measured whether a preview display defining the **Same** contour (Figure 1e) as the postsaccadic display (Figure 1g) would provide a benefit in response speed. Similarly, we assessed whether a **Different** (Figure 1f) preview contour could induce a performance cost. Note how the positions of the local elements never changed within one trial, even if the grouping cue and the contour displayed did. To account for the possibility that postsaccadic processing of Gabor elements could simply be selectively facilitated at the location of high-luminance preview elements, without involving a mid-level shape representation, we compared the Uniform baseline to a **Random** preview condition with less high-luminance elements (Figure 1c). Performance should then be worse in this condition. Finally, we investigated whether the mere presence of any preview object of roughly the same size and position could already partially explain performance benefits obtained following a Same preview, without relying on a detailed shape representation (**Localized** condition, Figure 1d).

Finally, we repeated the experiment while participants maintained steady fixation at the initial fixation point. The preview display was still presented at the peripheral screen location, whereas the test display was now presented foveally at the fixation location. In other words, in retinal coordinates the stimulus presentation sequence was comparable to the transsaccadic experiment, but the saccade itself was not performed. The aim of this control experiment was to account for the alternate possibility that receptive fields at the relevant level of representation would be large enough to cover both the presaccadic and the postsaccadic retinal stimulus position. Any effects observed would then not hinge critically on the execution of a saccade.

## Methods

### Participants

Six naïve observers and the first author performed the task. All participants had normal or corrected-to-normal eyesight. Ethical approval was given by the Ethics Board of the Faculty of Psychology and Educational Sciences of the University of Leuven, and written informed consent was obtained.

### Apparatus

An Iiyama Vision Master Pro541 CRT monitor with a viewable area of 17° by 13° was positioned 135 cm from the participants. It was configured to display visual stimulation at a spatial resolution of 800 by 600 pixels and a temporal resolution of 200 Hz. Phosphor persistence from ‘white’ (83.2 cd/m^2^) to ‘black’ (<0.01 cd/m^2^) luminance was found to be reduced to below 1% within 22 ms, compared to the average luminance measured over one refresh period of a white display presentation. All intrasaccadic display changes in the present study were performed at the start of the saccade; 99% of all saccade durations in the current experiment exceeded 30 ms. Moreover, all stimuli were rendered in shades of grey and never contained ‘black’ luminance values, further rendering any phosphor persistence from presaccadic to postsaccadic presentations irrelevant to visual perception [20]. Gamma correction and stimulus presentation were performed by a CRS Visage stimulus generator. Eye movement data were collected with a non-invasive dual-Purkinje Image eyetracker sampled at 1000 Hz, and processed using custom software on a Windows XP platform (manuscript submitted for publication). Subjects had two response buttons available, one for each hand.

### Stimuli

We aimed to produce displays consisting of spatially separated elements, which latently contained two different contours. The local feature information of these elements could then be manipulated to evoke each of these contours separately – or no contour at all in case of Uniform, Random, and Localized conditions. In presaccadic displays, the local elements were Gaussian blobs of which we manipulated the luminance. In postsaccadic displays, these local elements were replaced by Gabor patches of which we manipulated the orientation. The rationale for using identical local element positions between presaccadic and postsaccadic displays, irrespective of the preview condition, was to control for differences in element placement constraints between conditions.

Stimulus displays were created using GERT, the Grouping Element Rendering Toolbox (Demeyer & Machilsen, manuscript in preparation; see also [21]). First, a random closed contour description was created in polar coordinates using Radial Frequency Patterns [22]. Each contour consisted of 10 sinusoidal components with a random frequency between 2 and 4, a random amplitude between 0.03 and 0.1° (degrees of visual angle), and an entirely random phase. A fixed radius of 1° was added to the sum of these components to avoid the occurrence of extreme concavities or convexities. The centroid of the closed contour was then aligned to the center of the stimulus image to ensure a standardized saccade landing position [23]. Second, 100 new random closed contours were created using the same procedure, and superimposed on the first contour. Based on compatibility parameters such as the minimal segment length between contour intersections, the radial distances between both contours, and the angle of contour intersections, a second contour compatible with the first contour was selected. Third, local elements were positioned exactly on these contour descriptions. The average placement of elements was equidistant along the contour, but uniform jitter - again strictly along the contour – was added. This caused the actual element distance to randomly lie between 58% and 142% of the average element distance. A minimum distance of 50% of the average inter-element distance was respected between contour elements and contour intersection points. The remainder of the stimulus display was then filled randomly with local element positions until no proximity cue was present anymore (p>0.3). Fourth, a 3.8° by 3.8° display was rendered using these element positions. Preview images were rendered as collections of Gaussian blobs with a standard deviation of 0.05°. Against a background absolute luminance of 0.5, peak high luminance was 0.77 and peak low luminance was 0.64, where 1 would be the maximally attainable luminance by the monitor at the current resolution settings. Postsaccadic displays were rendered using oriented Gabor elements with a peak luminance of 0.77, a standard deviation of 0.06° to the Gaussian component, and a spatial frequency of 10 cycles per degree and phase of 0 to the sinusoidal component. Relevant contour elements were aligned along the local tangent of the contour description, with uniform random orientation jitter between 0 and plus or minus 22.5 degrees. All other elements - both those in the background and those defining the other contour latently contained within the display - had a completely random orientation. A 0.15° wide target square, consisting of a 2-pixel wide outer border at 0.75 luminance and a 3-pixel wide inside surface at 0.25 luminance, replaced a random background element. It was positioned at a maximal radius of 1.35° and a minimal radius of 0.75° from the center of the display. The target square was positioned inside both contours in 25% of the displays used, outside both contours in another 25%, and inside one but outside the other in the remaining 50%. These measures prevented the subject from using either the target eccentricity alone or a theoretical combination of the presaccadic contour information and the postsaccadic target position information to generate an accurate response. Postsaccadic grouping by collinearity was therefore necessary to solve the task.

In total, 80 different stimulus image sets were generated in this manner. The stimuli shown in Figure 1 constitute one such set. An intrafixation pilot experiment was performed on four subjects to select these sets from a greater collection of 160 sets, using the postsaccadic orientation-defined displays only. Specifically, we excluded sets that elicited too many incorrect or late answers (<75% correct responses), or deviant response speeds. Reaction times were normalized per subject to a mean of 0 and a standard deviation of 1, and pooled together per stimulus across subjects. We then removed all sets of which at least one stimulus display had an average normalized reaction time outside an interval of +−0.6 around the overall mean. Finally, we removed stimulus sets that based on a visual inspection were too similar to other sets.

### Procedure

**Figure 2:**
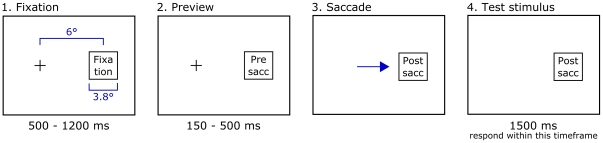
Procedure of the experiment. The observer fixates a fixation cross until the peripheral fixation display is replaced by the presaccadic stimulus. As soon as a saccade launch towards this preview is detected, it is replaced by the postsaccadic display. After saccade landing, a speeded response is required.

Figure 2 illustrates the experimental procedure. In a dimly lit room, participants were instructed to fixate a cross to the left of the center of the screen, while 6° to the right of the cross an apparently unstructured display filled with low-luminance Gaussian elements was shown (Figure 1a). From this image alone, no contour grouping could take place. After a button press a random fixation period of 500 to 1200 ms started, followed by a sudden luminance increase in some or all of the Gaussian elements (Figure 1b–f). Participants were then required to make an immediate saccade towards the center of the peripheral display, within 150 to 500 ms. As soon as the eye moved outside the fixation zone, the local elements retained their positions but changed into oriented Gabor elements defining a closed contour (Figure 1g–h). One of the presaccadic elements was instead replaced by a target square. Participants were to respond within 1500 ms whether this target was located inside (left button) or outside the contour (right button). Reaction times were measured from the onset of the Gabor test display onwards. No mask was present after stimulus presentation and no feedback was given, except during the first 20 trials. These were intended as practice and were not included in any analysis or statistic. The median saccadic latency on successfully executed trials was 213 ms across all subjects, with the 10% and 90% percentiles at 182 and 287 ms, respectively.

The experimental conditions were created by increasing the luminance in different subsets of the Gaussian preview elements, visible during the saccadic reaction time period only. We lit up either all elements (Uniform condition), only those elements situated exactly on an object contour (Same and Different conditions), half the elements across the entire display (Random condition), or half the elements within the maximal radius of both latent object contours (Localized condition). All conditions were randomly intermixed on a trial-by-trial basis.

Per participant, 500 trials were collected across 10 blocks. Trials aborted due to an incorrect saccade were recycled after each block; twice-aborted trials were not recycled again. Late or incorrect responses were never recycled. In total, 5.03% of all trials were lost.

Three subjects were newly recruited for the intrafixation control experiment, four had also participated in the main experiment. The procedure differed on two aspects only. First, the Gabor test display was not presented at the same location as the Gaussian preview display but at the initial fixation location, where subjects were required to maintain fixation throughout the trial. Second, the replacement of the Gaussian by the Gabor display was not contingent on the start of a saccade. Instead, the exposure duration of the Gaussian preview display was set to a random value between 175 and 275 ms. In total, 0.06% of all trials were lost.

## Results

### Eye movement analysis

**Figure 3:**
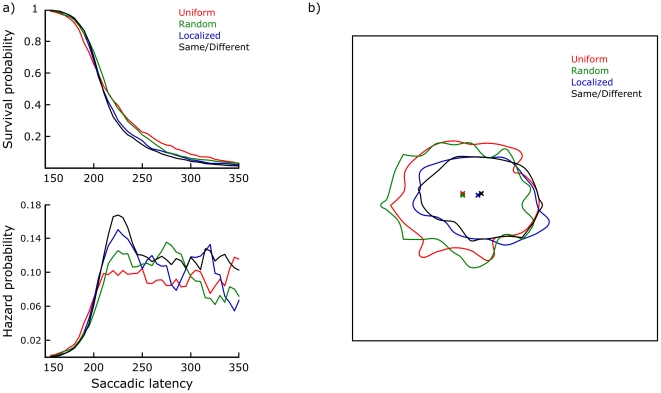
Eye movement data. a) Survival and hazard probability plots of saccadic latencies, per condition. b) Saccade landing positions within the boundaries of the stimulus image, per condition. These iso-frequency contours enclose 90% of all saccade landings in each condition. It can be seen that Localized and Same/Different preview images elicit both faster and more accurate saccades.

First, the eye movement data were considered. For these analyses the Same and Different conditions were treated as identical, since they could not be distinguished between based on the preview display alone. Saccadic latencies - preview durations - only differed significantly in an ANOVA when contrasting Uniform and Random conditions with Localized and Same/Different conditions (*F*(1,24) = 6.99, *p* = 0.01). However, the effect size was small: The former conditions were on average 7 ms slower in eliciting saccades, despite having a greater total luminance increase at the onset of the preview display. Figure 3a illustrates the saccadic latency distributions by means of a discrete time survival analysis [24]–[25]. This analysis was performed after normalizing each subject's median to the overall median, then pooling the data across subjects. Bins of 5 ms were used; an X-axis range including 97.7% of all data points is shown here. The top figure plots the survival function for each condition, i.e. the probability that no saccade has started yet. The survival probability equals one minus the cumulative proportion. The bottom figure plots a smoothed hazard function for each condition, i.e. the conditional probability that a saccade starts within each time bin given that it has not already started. Smoothing was done through a five-bin moving average. Essentially, these hazard functions reflect the dynamic evolution of saccade initiation forcing. There appears to be an early advantage for Uniform previews (150–200 ms), probably based on their higher overall luminance increase compared to the fixation display. The intermediate period (200–250 ms) is clearly critical for saccade initiations based on object-like stimulation, i.e. Localized and especially Same/Different previews. Random previews are slowest to begin eliciting saccades, but then do so within a somewhat more constrained time window than Uniform previews. Figure 3b plots iso-frequency contours for the distributions of landing positions, enclosing 90% of all saccade landings in each condition. The borders of the figure coincide with the border of the stimulus image. The contours shown are based on a 2D histogram using 10 pixel (0.21°) bins. The combined marginal median landing position is also marked for each condition. ANOVAs on these data revealed a significant difference in mean landing position when contrasting Uniform and Random conditions with Localized and Same/Different conditions (*F*(1,24) = 168.42, *p*<0.01). A similarly significant difference was found when comparing the spread of saccade landing positions using an Analysis of Variance of Variance [26]: *F*(1,24) = 149.36, *p*<0.01. In addition, landings on Localized preview trials were significantly different from landings on Same/Different preview trials (*F*(1,24) = 4.73, *p* = 0.04).

We then used the eye movement data to identify and remove trials where subjects had difficulties executing the task. Trials with very inaccurate saccades (landing position >1.5° from the stimulus center, 0.69% of all completed trials) or anomalously long-lasting saccades (>85 ms, 0.33% of all completed trials) were removed. Subjects almost always made additional saccades towards the target square before responding (99.31% of all completed trials). The data on these additional saccades therefore provide us with a measure of how easily subjects could locate the target square. Importantly, there was no difference in initial postsaccadic fixation duration between the different preview conditions (mean = 210 ms, *F*<1). This indicates that the preview condition did not affect the speed with which subjects could locate the target square within the postsaccadic display. To improve the quality of the dataset, we censored those trials where subjects apparently had difficulties finding the target. Trials with more than two additional saccades (2.65% of all completed trials) or exactly two additional saccades and a final fixation duration equal to or greater than 240 ms (11.76% of all completed trials) were removed. Trials with exactly two additional saccades and a fixation duration below 240 ms were considered to have generated the manual response without actually requiring the second additional saccade. The cut-off point of 240 ms was selected so that the median total reaction time on these trials (653 ms) was equal to that of trials with only one additional saccade. In comparison, trials with exactly two additional saccades and a final fixation duration above 240 ms were much slower to elicit a response (median = 841 ms). In total, 84.90% of all completed trials were retained. These trials can be considered uniform in their execution of the main saccade and localization of the target square after the main saccade landing. The different preview conditions were similarly represented in the final data set: It consisted of 19.31% Uniform, 18.75% Random, 20.45% Localized, 20.66% Same and 20.84% Different trials.

### Perceptual effects

**Figure 4:**
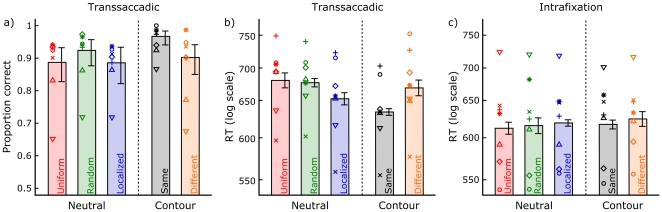
Main results. Panel a) displays the proportion correct and Panel b) the reaction time results for the transsaccadic experiment. c) Reaction time results for the intrafixation control experiment. Error bars denote 95% confidence intervals of the effect variability across subjects. That is, the standard error for each condition was computed as the standard deviation of the subjects' mean score for that condition relative to their overall mean score, divided by the square root of the number of subjects. Different symbols illustrate the performances of different participants. The first author's data is in all three graphs symbolized by the circular marker; the markers of the other subjects correspond to one another in the first two graphs only.

Figure 4a shows the accuracy results for the manual responses. In a within-subjects logistic regression analysis, only the Same condition was found to be significantly more accurate than the Uniform baseline (*β_Same_* = 1.40, *t*(1962) = 4.13, *p*<0.01). Reaction times were only analyzed for correct responses, after performing a log-transform to reduce the asymmetry in their distributions. Saccadic latency was not included as a covariate, since the size and the direction of the differences between conditions did not allow for an alternate explanation of the data in terms of preview duration. Saccade landing accuracy did pose an important confound to the interpretation of the data, and was therefore included as a covariate. Indeed, it was a significant predictor of manual reaction times (*F*(1,6) = 7.60, *p* = 0.03). No heterogeneity of slopes between conditions was present (*F*<1). Crucially, a significant effect of preview condition on top of the effect of the landing accuracy covariate was found (*F*(4,24) = 9.85, *p*<0.01). Figure 4b shows the reaction time results on a log scale, corrected for landing accuracy. Localized neutral previews elicited faster responses when contrasted with Uniform and Random previews (*F*(1,24) = 7.00, *p* = 0.01), which did not differ (*F*<1). Same trials were responded to 35 ms faster than Different trials (*F*(1,24) = 27.42, *p*<0.01). Relative to the Localized neutral preview baseline, this amounted to a 16 ms benefit and a 19 ms cost, respectively (both *p* = 0.02). Figure 4c shows the results of an analogous reaction time analysis for the intrafixation control experiment. No significant difference existed between the various preview conditions (*F*<1). Since the average percentage correct was very high in all conditions (between 95% and 97%) and for all subjects (between 94% and 98%), we did not generate a figure for the accuracy results.

**Figure 5:**
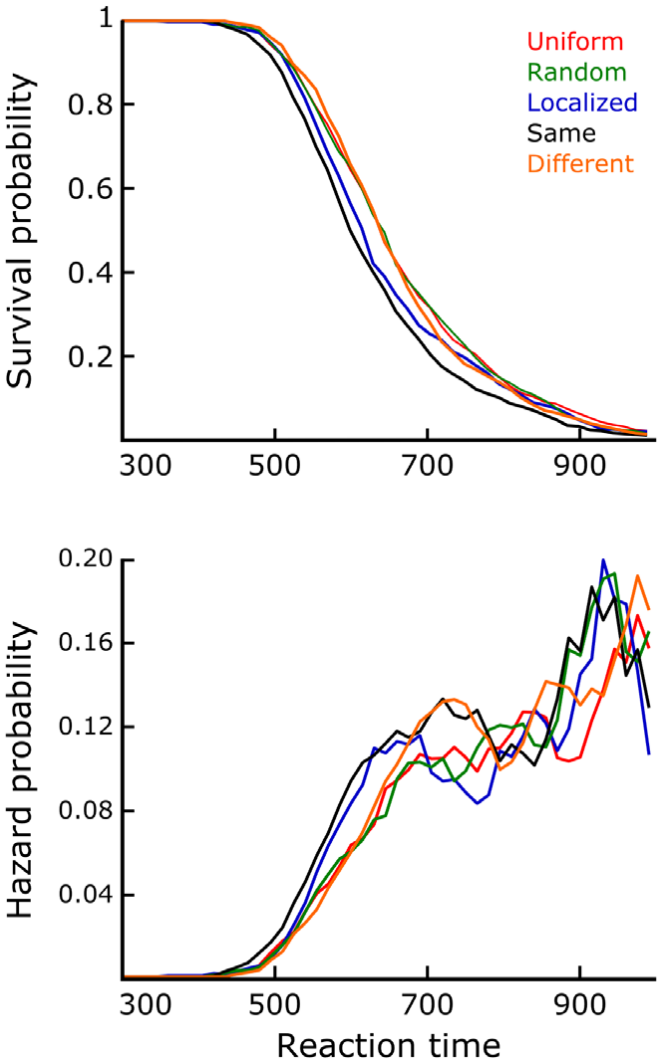
Survival analysis of the main results. Translating the manual reaction times of the transsaccadic experiment into survival and hazard probabilities, it can be seen that the mean pattern of Figure 4b is mainly contained within the fastest half of the responses.

Figure 5 shows the results of a discrete time survival analysis of the manual reaction times of the transsaccadic experiment. Again individual subject medians were normalized to the overall median before pooling the data. To correct for the effect of landing accuracy, each data point was linearly regressed to a landing on the center of the display. Analogous to Figure 3a, this figure shows the survival and smoothed hazard functions for each condition. 15 ms bins were used; the X-axis range displayed here includes 98.35% of all data. The hazard functions show that the pattern of results expressed by Figure 4b is mainly contained within the fastest responses. Responses slower than around 650 ms (at which point 44.64% of all trials still ‘survive’) show a more complicated pattern, where the mean effects as reported can no longer be observed. Note that in a model data set where the distributions of all conditions would be normal with equal variance, the mean pattern would remain present in the hazard functions throughout the reaction time interval. This stresses the importance of using an easy, automatic task to assess preview effects on perceptual grouping speed.

### Coarse shape properties

One could speculate that the slower responses on Different trials are attributable to differences in shape properties at the coarsest level of description only, i.e., vague blobs with a sometimes conspicuous aspect ratio and orientation. We here define these properties as the aspect ratio and orientation of the smallest possible bounding box rectangle around each contour. Looking only at trials where the pre- and postsaccadic shapes were elongated (both aspect ratios greater than 1.2, corresponding to 21.21% of all Different trials included in the reaction time analysis), there was a marginally significant correlation between the Different preview log-transformed reaction times and the size of relative orientation differences (*r* = 0.14, *t*(6) = 2.33 *p* = 0.06). However, removing trials with a considerable orientation difference (>33°) reduced the overall cost compared to the Localized condition by less than 10%. The actual impact of differently oriented elongated shapes on reaction times in the Different condition is therefore minimal. Moreover, a difference in aspect ratio did not explain the Different results either: On Different trials where at least one of both shapes was not elongated (aspect ratio <1.2) or both were elongated but no large orientation difference was present (<33°), no significant correlation existed between reaction times and the relative aspect ratios of pre- and postsaccadic stimuli (*r* = 0.01, *t*(6) = 0.20, *p* = 0.85). Thus, coarse shape properties were not relevant when using the current stimulus set.

## Discussion

Two processes contributing to transsaccadic object perception are apparent.

First, a peripheral glimpse of any localized form, even if only vaguely defined, induced faster postsaccadic object grouping behavior when compared to uniform or entirely random displays. This finding agrees with previous reports documenting that the presaccadic presence of any object at or around the saccade target location is by itself relevant for postsaccadic vision [27]–[28]. The observation that saccade execution was in addition both faster and more accurate given a localized preview indicates that subjects indeed processed the presaccadic form information and involved it in saccade programming, despite the predictability of the optimal saccade landing position. This adds to the body of evidence suggesting that human saccadic behavior is fundamentally object-based [29]–[30]. For our current purposes, however, the main implication is that a suitable baseline to compare effects of more detailed transsaccadic object shape information with would have to be object-like (Localized) rather than object-less (Uniform).

Second and most crucially, additional effects of the transsaccadic congruency of object shapes were observed: Same preview displays provided a benefit in reaction times, compared to the Localized condition, whereas Different preview displays slowed down object contour grouping after the gaze shift. This implies that perceptually grouped shape information was carried across the saccadic eye movement, and was subsequently employed in organizing the postsaccadic display and establishing a foveal shape percept.

The reaction time effects were not based on an image-like representation, since they occurred despite a change in the relevant grouping cue. This suggests that transsaccadic vision makes abstraction of both the local image information itself and the specific grouping principle through which it defines object contours. The alternate hypothesis proposed, that greater local facilitation might occur at the position of high-luminance preview elements, can be discarded given the absence of any difference between the Uniform and Random preview conditions. Indeed, the irrelevance of low-level luminance or contrast information for transsaccadic vision has previously been established by other authors [5], [14], [31].

Differences in coarse shape properties between pre- and postsaccadically presented objects explain only a small part of the preview costs observed. This is probably due to the uniformity of the stimulus set used. However, as the absence of accuracy costs for Different trials shows, the postsaccadic grouping cues could always override the incorrect shape suggested by the preview; a reaction time cost was their only measurable effect. We propose that the reaction time costs that are present in the Different preview condition are to be attributed to a time-consuming reorganization of the grouping topography before another perceptual organization can be inferred from the visual input. For only subtly different perisaccadic stimuli, we have previously found that integration of object shapes into an intermediate percept can take place [12]. Mid-level object shape representations could then constitute a common representational ground to achieve this across the different spatial resolutions of the visual field.

In the intrafixation control experiment we found no difference between any of the conditions. This suggests that the present data cannot merely be explained by a sufficiently large receptive field size at the relevant level of representation. The execution of a saccade towards a spatiotopically stable stimulus appears to be a necessary condition to observe preview effects of perceptually grouped shapes, at least across the retinotopic stimulus distance used in the present study.

### Persistence and remapping of perceptual organization

The intermediate nature of transsaccadic visual memory, which we suggest here, is not only supported by a combined logic of representational commensurability and processing efficiency but also by prior empirical findings.

On a behavioral level, within-fixation visual memory for objects has been shown to contain a high-capacity component at intermediate levels of visual processing, persisting at least several hundred milliseconds after stimulus offset [32]–[34]. For instance, the study of Landman et al. [33] demonstrated that visual memory for figure-ground segregated objects remains unaffected by a homogenous intervening mask display, whereas performance fell when another figure-ground segregated stimulus followed. The link to the current results is clear: High-capacity representations of perceptual organization can persist long enough to bridge the saccadic interruption (which typically lasts less than 100 ms). Importantly, Germeys et al. [13] recently demonstrated that indeed such detailed and volatile visual memory traces can also be retained across saccadic eye movements, to be used in a transsaccadic change detection task.

Neurophysiological evidence corroborates the idea that the visual system dispenses with local feature information when a more global analysis of the input is reached. This occurs even in the absence of eye movements: An inverse correlation exists between early visual activation levels and either the degree of image structure [35] or the amount of activation in the lateral-occipital complex [36]. The latter finding could be especially relevant, as it concerns a cortical area related to cue-invariant shape perception [37]–[38].

The visual cortex is fundamentally organized around the retinal projection locations of objects, and gaze shifts change these projection locations. The spatiotemporal continuity of visual representations would therefore seem to be compromised during scene scanning. As a solution, it has been proposed that neurons remap their receptive fields prior to a saccade [39]. This receptive field remapping has been suggested to be the driving force behind a plethora of perisaccadic empirical phenomena, including adaptation after-effects, the temporal development of inhibition of return, location judgments, and time offset judgments [14], [40]–[42]. Interestingly, however, remapping is more prevalent in mid- than in low-level visual areas [14], [43]. Moreover, in a comprehensive neuro-computational implementation of perisaccadic perception, Hamker, Zirnsak, Calow, and Lappe [44] recently showed that the populations of neurons relevant to behavioral remapping phenomena need a certain minimal receptive field size, and therefore cannot be situated early in the visual stream. These observations all lend further support to the primacy of more complex, mid-level visual areas in transsaccadic vision.

### Beyond the saccade target object?

Gaze shifts are closely linked to attention shifts [45]. In the present study, as in many natural situations, the probable locus of attention always coincided with both the task-relevant stimuli and the saccade target location. No firm conclusions regarding the unique contribution of attention mechanisms to the current results can therefore be drawn. It is worth noting though that high-capacity visual memory persistence, both within fixations and across saccades, is often thought of as being pre-attentive [13]. This has important theoretical implications for the way in which humans process and explore scenes: Multiple object shape representations could then be retained in parallel across each saccade, instead of the saccade target object only. Similarly relevant, recent studies have shown that the grouping and figure-ground segregation processes giving rise to persistent mid-level representations do not strictly require focused attention either [46]–[47]. But, there is as of yet no evidence that this pre-attentive formation and persistence of segregated shapes also extends to the type of functional benefits that we have demonstrated in the current study. Certainly, the present results open up interesting research opportunities regarding scene segmentation across a series of saccades.

### Conclusions

We conclude that following a gaze shift, object perception processes utilize the perceptual organization that was inferred from the peripheral preview of an object, independently of how it was defined exactly in the presaccadic image. In general, the current results emphasize that real-life object perception is not an intrafixation phenomenon, as it is often studied in experimental settings, but instead relies on signals generated from previous fixations to organize its visual input [48]. Pooling its information in this way, the brain is demonstrated to be a three-way integration device: Across retinal space, perisaccadic time, and grouping cues.
